# Muscle Involvement in a Large Cohort of Pediatric Patients with Genetic Diagnosis of Mitochondrial Disease

**DOI:** 10.3390/jcm8010068

**Published:** 2019-01-10

**Authors:** Cristina Jou, Juan D. Ortigoza-Escobar, Maria M. O’Callaghan, Andres Nascimento, Alejandra Darling, Leticia Pias-Peleteiro, Belén Perez-Dueñas, Mercedes Pineda, Anna Codina, César Arjona, Judith Armstrong, Francesc Palau, Antonia Ribes, Laura Gort, Frederic Tort, Placido Navas, Eduardo Ruiz-Pesini, Sonia Emperador, Ester Lopez-Gallardo, Pilar Bayona-Bafaluy, Raquel Montero, Cecilia Jimenez-Mallebrera, Angels Garcia-Cazorla, Julio Montoya, Delia Yubero, Rafael Artuch

**Affiliations:** 1 Clinical Biochemistry, Pathology, Paediatric Neurology and Molecular Medicine Departments and Biobank, Institut de Recerca Sant Joan de Déu and CIBERER-ISCIII, 08950 Esplugues, Spain; cjou@sjdhospitalbarcelona.org (C.J.); jortigoza@sjdhospitalbarcelona.org (J.D.O.-E.); mocallaghan@sjdhospitalbarcelona.org (M.M.O.); anascimento@sjdhospitalbarcelona.org (A.N.); adarling@sjdhospitalbarcelona.org (A.D.); lpias@sjdhospitalbarcelona.org (L.P.-P.); belen.perez@vhir.org (B.P.-D.); pineda@sjdhospitalbarcelona.org (M.P.); acodina@fsjd.org (A.C.); carjona@fsjd.org (C.A.); jarmstrong@sjdhospitalbarcelona.org (J.A.); fpalau@sjdhospitalbarcelona.org (F.P.); rmontero@sjdhospitalbarcelona.org (R.M.); cjimenezm@fsjd.org (C.J.-M.); agarcia@sjdhospitalbarcelona.org (A.G.-C.); rartuch@sjdhospitalbarcelona.org (R.A.); 2 Secció d’Errors Congènits del Metabolisme—IBC, Servei de Bioquímica i Genètica Molecular, Hospital Clínic, IDIBAPS, CIBERER-ISCIII, 08028 Barcelona, Spain; ARIBES@clinic.cat (A.R.); LGORT@clinic.cat (L.G.); FTORT@ciberer.es (F.T.); 3 Centro Andaluz de Biología del Desarrollo, Universidad Pablo de Olavide and CIBERER-ISCIII, 41013 Sevilla, Spain; pnavas@upo.es; 4 Departamento de Bioquímica, Biología Molecular y Celular, Instituto de investigación Sanitaria de Aragón and CIBERER-ISCIII, Universidad de Zaragoza, 50013 Zaragoza, Spain; eduruiz@unizar.es (E.R.-P.); seortiz@unizar.es (S.E.); esterlop@unizar.es (E.L.-G.); pbayona@unizar.es (P.B.-B.); jmontoya@unizar.es (J.M.)

**Keywords:** mitochondrial diseases, myopathy, pediatric patients, biochemical markers, muscle histopathology, next generation sequencing

## Abstract

Mitochondrial diseases (MD) are a group of genetic and acquired disorders which present significant diagnostic challenges. Here we report the disease characteristics of a large cohort of pediatric MD patients (*n* = 95) with a definitive genetic diagnosis, giving special emphasis on clinical muscle involvement, biochemical and histopathological features. Of the whole cohort, 51 patients harbored mutations in nuclear DNA (nDNA) genes and 44 patients had mutations in mitochondrial DNA (mtDNA) genes. The nDNA patients were more likely to have a reduction in muscle fiber succinate dehydrogenase (SDH) stains and in SDH-positive blood vessels, while a higher frequency of mtDNA patients had ragged red (RRF) and blue fibers. The presence of positive histopathological features was associated with ophthalmoplegia, myopathic facies, weakness and exercise intolerance. In 17 patients younger than two years of age, RRF and blue fibers were observed only in one case, six cases presented cytochrome c oxidase (COX) reduction/COX-fibers, SDH reduction was observed in five and all except one presented SDH-positive blood vessels. In conclusion, muscle involvement was a frequent finding in our series of MD patients, especially in those harboring mutations in mtDNA genes.

## 1. Introduction

Of all inherited metabolic diseases, mitochondrial diseases (MD) occur at the highest frequency (incidence around 1/5000 births) [1]. The double genetic control of the oxidative phosphorylation (OXPHOS) pathway (controlled by nuclear DNA (nDNA) and mitochondrial DNA (mtDNA)) means that it is possible to inherit MD through either mode of inheritance [1]. Furthermore, the intricate and fundamental metabolic pathways occurring in mitochondria leads to, amongst other problems, a remarkable difficulty reaching an etiological diagnosis of MD [2]. Genotype-phenotype correlations for MD are not well-established because mutations in the same gene can cause varied clinical phenotypes (allelic heterogeneity) and similar clinical pictures can be caused by mutations in different mitochondrial genes (locus heterogeneity) [1,3]. Furthermore, genetic disturbances in other non-mitochondrial pathways may mimic MD or even cause a secondary mitochondrial dysfunction [3].

From a clinical point of view, there are classical and well-recognized mitochondrial syndromes that have been extensively reported [1,4]. In these cohorts of patients, mutations either in nDNA or mtDNA genes are the cause of the disease. In other cases, clinical pictures may be incomplete and unspecific, especially at pediatric age, increasing diagnostic difficulties [1,4]. In most pediatric patients, the clinical signs may be considered severe or even life-threatening, especially when MD appears early in life. High-energy demanding tissues are affected first, with neuromuscular involvement being one of the cardinal features of MD [5,6].

At present, a MD diagnosis can require extensive non-invasive and invasive tests including clinical and biochemical studies, imaging, neurophysiology and muscle pathology assessment, and, as a final step, definitive molecular genetic confirmation [4]. A biochemical analysis for MD includes the measurement of lactate, pyruvate, amino acids, FGF-21, and GDF-15 in blood and organic acids in urine, as well as routine parameters such as creatine kinase (CPK) to assess muscle involvement. However, even when used in combination, neither high specificity nor high sensitivity is expected [4,7]. Cerebrospinal fluid analysis of lactate and other biomarkers may improve the diagnostic yield of blood tests [8]. Other biomarkers, such as methylglutaconic, thymidine, or coenzyme Q10, may provide further insights to elucidate furthering the aetiology of MD [7]. A muscle biopsy may be required to complete the diagnostic process since various typical features of the muscle can be detected even in the absence of clinical muscle involvement, and it may be of paramount importance in the diagnostic workflow [1,4].

Although the advent of next-generation sequencing techniques (NGS) is changing the diagnostic paradigm [9,10,11,12,13,14,15,16,17,18], a remarkable percentage of patients (around 50% in most series) remain undiagnosed, or they are ultimately diagnosed with other non-mitochondrial conditions. NGS has facilitated diagnosis, and several recently reported patient series associated more than 290 genes as causative of MD [19]. Different genetic approaches have been applied, including targeted mitochondrial gene panels, whole exome (WES) and whole genome sequencing (WGS) and, recently, the combined genetic analysis of both mtDNA and nDNA [20,21]. As a result, not only is diagnostic efficacy enhanced, but new pathomechanisms are being unraveled, and unexpected findings are being recognized [17].

Taking the above details together, our overall goal is to report the disease characteristics of a large cohort of pediatric MD patients with a definitive molecular diagnosis, with a special emphasis on clinical muscle involvement and biochemical, histopathological, and genetic features.

## 2. Material and Methods

### 2.1. Patients

Over the last 25 years, we have recruited a cohort of 108 pediatric patients with MD who have visited Hospital Sant Joan de Déu. This study was approved by the local Ethics Committee (protocol number PIC-149-17) and performed in accordance with the Declaration of Helsinki. All participants signed an informed consent. Of this initial cohort of patients, 13 were excluded from the study because no clinical, biochemical, or histological data were available in the clinical records. This was because they were patients who visited only once and follow-up was not possible, or because they were diagnosed after an acute presentation with a fatal outcome. Large family cohorts (for example the MELAS (mitochondrial encephalopathy, lactic acidosis and stroke-like episodes) patients previously reported by our group [22]) were not included in the study. Thus, a final cohort of 95 patients were included (age range, between 1 day and 16 years, average age of 4.0 years, standard error of the mean (SEM) = 0.45; sex distribution 40 females and 55 males). The main clinical details, and biochemical, histopathological, and molecular data are reported in Table S1.

### 2.2. Methods

#### 2.2.1. Patient Recruitment

MD patients are usually diagnosed with a combination of clinical, biochemical, and histopathological data, when the genetic bases of the disease are elusive [1]. In this work we recruited MD patients only when genetic diagnosis was positive, and they were further classified depending on the origin of mutation (nDNA (*n* = 59) or mtDNA (*n* = 44)). This classification is relevant since the clinical phenotype (including muscular involvement), the way of inheritance, the familial antecedents and other features are different in mtDNA and nDNA patient groups. For the patient selection, we included genes with a primary role specific to OXPHOS biogenesis and genes with a secondary impact on OXPHOS biogenesis as well as other cellular functions, as reported by Frazier et al. [19]. The biological functions of these genes include: (1) OXPHOS subunits, assembly factors and electron carriers; (2) mtDNA maintenance; (3) mtDNA expression; (4) enzyme cofactors; (5) mitochondrial homeostasis and quality control; and (6) general energy metabolism [19]. In Table S1, the different molecular mechanisms associated with the mutated genes are stated.

#### 2.2.2. Clinical Data

Data collected from medical records included age of presentation, gender, survival (age of death) and specific muscular signs and symptoms such as ophthalmoplegia, myopathic facies, exercise intolerance, weakness, rhabdomyolysis and myopathic electromyography (EMG). Data were available from 78 patients.

#### 2.2.3. Samples

Blood, urine, and when indicated, muscle or other tissue biopsies were collected for diagnostic purposes and stored in the Hospital Sant Joan de Déu Biobank. DNA from blood and muscle samples was isolated using standard procedures. Samples were taken in accordance with the 2013 revised Helsinki Declaration of 1964. Parental informed consent forms were obtained in all cases. The ethical committee of Sant Joan de Déu Hospital approved the study.

#### 2.2.4. Biochemical Analysis

Blood lactate, pyruvate, amino acids (alanine), FGF-21/GDF-15, CPK and urine organic acids (lactate and Krebs cycle metabolites) were analyzed as previously reported [7,23] in 89 patients. Hyperlactacidemia was only considered when 2 or more blood lactate levels were documented (the same criterion was used for pyruvate and alanine) [1].

#### 2.2.5. Histopathological Analysis

Sixty muscle biopsies were studied (average age at biopsy was 5.86 years; SEM = 0.78). All muscle biopsies were analyzed by an experienced neuropathologist. Each muscle specimen was collected from quadriceps and processed and stained according to standard protocols with modified Gomori trichrome, succinate dehydrogenase (SDH), cytochrome c oxidase (COX) and oil-red O stain for lipids [4]. The following muscular pathological features were recorded: ragged red fibers (RRF), fibers with increased SDH staining (blue fibers), COX negative fibers (COX-), COX reduction, SDH reduction and SDH-positive blood vessels. To compare histopathological features of MD infant patients (younger than 2 years old), we assessed the presence of COX-, RRF/blue fibers, SDH reduction, SDH-positive blood vessels and lipid increase (1) in 17 cases selected from the whole cohort of patients. As a control group, we studied 12 patients with different congenital myopathies: nemalinic (*n* = 5), myotubular (*n* = 2), central core (*n* = 1), merosin-deficient congenital muscular dystrophy (MDC1A) (*n* = 3) and congenital myotonic dystrophy type 1 (DM1) (*n* = 1).

#### 2.2.6. Genetic Analysis

Because the patients were studied over the past 25 years, different approaches were applied for genetic diagnosis. The mtDNA mutations were studied by specific procedures, including SANGER sequencing, Southern-blot, and real-time polymerase chain reaction (PCR), as previously reported [22]. Depending on the suspected causative gene, nuclear DNA was analyzed either by SANGER sequencing, or, over the past 4 years, by NGS, with customized [24] or commercial panels (TruSight One Sequencing Panel, Illumina (San Diego, CA, USA)) using MiSeq and NextSeq500 sequencers (Illumina). Whole exome sequencing was only performed in selected cases. To test the nuclear mutations detected, SANGER sequencing was done in all cases. Progenitor studies, in-silico analysis or functional-cellular studies were carried out to evaluate the inheritance model and to confirm the pathogenicity of genetic variants, when required.

#### 2.2.7. Statistical Analysis

We categorized the clinical, biochemical and histological data as normal or impaired (see Table S1). Statistical associations among the different categorical variables were studied with the chi-square test using the SPSS 20.0 program (IBM, Armonk, NY, USA).

## 3. Results

**Figure 1:**
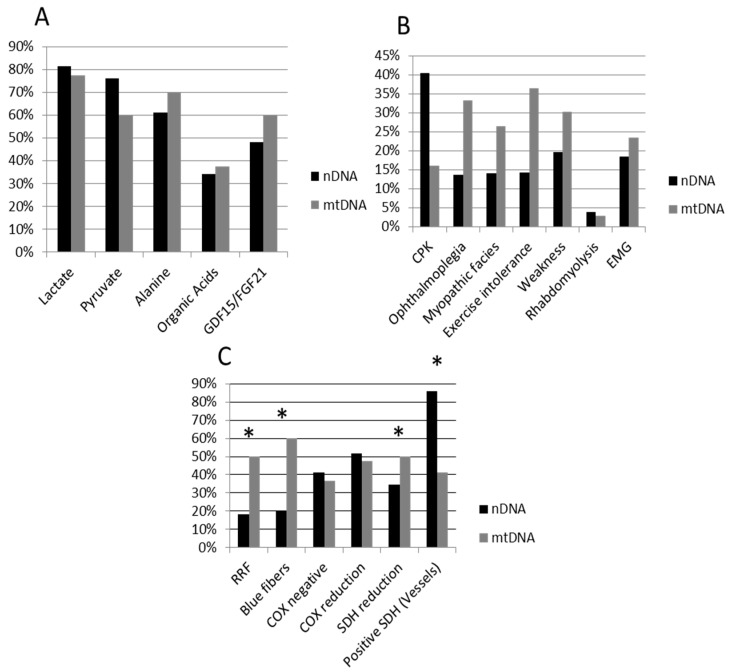
Percentage of impaired biomarkers (**A**), muscular involvement (**B**) and positive histopathological features (**C**) in nDNA (black bars) and mtDNA (grey bars) patients. Whiskers indicate significant differences (chi-square test) between nDNA and mtDNA groups. nDNA patients showed significantly higher frequency of SDH reduction and SDH-positive vessels, while mtDNA patients shower a higher frequency of RRF and blue fibers, and near significative for ophthalmoplegia and exercise intolerance. nDNA, nuclear DNA; mtDNA, mitochondrial DNA; CPK, creatine phosphate kinase; EMG, myopathic electromyography; RRF, ragged red; SDH, succinate dehydrogenase; COX, cytochrome c oxidase.

Muscular clinical signs, and biochemical and histopathological data for the whole patient cohort and for the mtDNA and nDNA subgroups are presented in Table 1 and Figure 1. A total of 51 patients harbored mutations in nDNA and 44 patients had mutations in mtDNA. Age was significantly lower in the nDNA group (average: 2.7 years, SEM 0.45) when compared with the mtDNA group (5.4 years, SEM 0.76) (Mann-Whitney U-test *p* < 0.0001). A higher frequency of nDNA patients had muscle SDH reduction and SDH-positive vessels, while a higher frequency of mtDNA patients had RRF, blue fibers, and nearly significative for ophthalmoplegia and exercise intolerance (Table 1, Figure 1).

We classified the 95 patients according to their clinical features (presence or absence of myopathy) and to histopathological features (RRF, blue or COX-fibers, positive or negative) (Table 2). As expected, the presence of muscular involvement was associated with positive RRF, blue and COX-fibers. Moreover, positive histopathological features were associated with ophthalmoplegia, myopathic facies, weakness and exercise intolerance. No associations were observed in these two groups for other variables, including the biochemical markers.

Muscle biopsies were concomitantly analyzed in 15 cases for serum GDF-15 and FGF-21 levels. There were 6 cases with normal FGF-21/GDF-15, and neither RRF nor blue fibers were detected (there were only two COX-fiber cases). In the 9 cases showing high FGF-21/GDF-15 values, RRF were observed in four cases, blue fibers in five cases and COX-fibers in seven cases.

**Figure 2:**
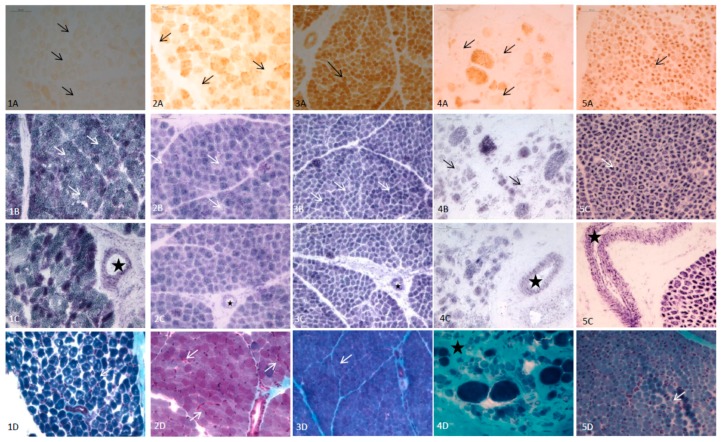
Main histopathological features (quadriceps muscle) of 5 infants with mitochondrial disease (MD) and other congenital myopathies. (1) Patient with AGK mutations (3 days old); (**1A**): Total absence of COX (arrows). (**1B**): Normal stain of SDH (arrows). (**1C**): SDH-positive vessels (star). (**1D**): Modified Gomori trichrome: No RRF (arrow marks a normal fiber). (2) Patient with SUCLA2 mutations (6 months old); (**2A**): Isolated COX negative fibers (arrows). (**2B**): Mild reduction of SDH (arrows). (**2C**): SDH-positive vessels (star). (**2D**) Modified Gomori trichrome: occasional subsarcolemal mitochondrial proliferation (arrows). (3) Patient with MT-ATP6 mutations (1 month old); (**3A**): COX: Muscular fibers with a normal pattern showing the highest intensity of stain in type I fibers, and pale and intermediate intensity in type II fibers (arrow mark a type I muscular fiber). (**3B**): Muscular fibers with normal SDH stain. (**3C**): SDH-positive vessels (star). (**3D**): Muscular fibers with normal pattern and no RRF with modified Gomori trichrome stain. (4) Patient with LAMA2 mutations (6 months old) (**4A**): Isolated fibers with COX reduction consistent with pre-necrotic fibers (arrows). (**4B**): Isolated fibers with SDH reduction (arrows). (**4C**): SDH-positive vessels (star). (**4D**): Modified Gomori trichrome; marked increase of endomysial tissue (star) consistent with dystrophic pattern, no RRF. (5) Patient with miotubularin mutation (1 month old). (**5A**): No COX negativefibers (arrows). (**5B**): No reduction of SDH (arrows). (**5C**): SDH-positive vessels (star). (**5D**): Modified Gomori trichrome. Large central nuclei in several fibers (arrows). No RRF.

In the whole cohort of patients, the most frequent histopathological finding was SDH-positive vessels, followed by COX reduction/COX-fibers (Table 1). In the 17 patients that were less than 2 years of age, RRF and blue fibers were observed only in one case (mutations in TK2), six cases presented COX reduction/COX-fibers (mutations in *AGK, PUS1, KARS, SUCLA2, MT-ATP6* and *TK2*) and SDH reduction was found in five patients (mutations in *PUS1, ECHS1, KARS, MT-ATP6* and *COX7*). All of them, with one exception, presented SDH-positive blood vessels. Lipids were increased in 9 out of 10 biopsies analyzed. In the 12 cases with congenital myopathies, no RRF/blue fibers were observed and SDH reduction was detected only in one case (DM1). We observed COX reduction in four cases, corresponding to MDC1A (two cases), DM1 (one case) and nemaline myopathy (one case). SDH-positive blood vessels were detected in all of these 12 cases. No increased lipids were observed in any of them. In Figure 2, the main histopathological features in selected infants with MD and non-mitochondrial congenital myopathies are shown.

The number of genetic diagnoses (nDNA) has significantly increased within the last 4 years due to the application of NGS techniques. In the 2015–2018 period, from the whole cohort of patients, a total of 26 cases harboring nDNA mutations were solved by NGS. There are still some unsolved cases (Table S1), corresponding to patients with mtDNA depletions or multiple deletions where the molecular bases are under investigation (nDNA).

## 4. Discussion

Here, we report one of the largest cohorts of pediatric patients with a molecular diagnosis of a MD. While most of the previous works have reported on the diagnostic yield of NGS techniques in MD patients [9,10,11,12,13,14,15,16,17,18,19,20,21], in this study we focused our attention on muscular features of pediatric MD patients with a definitive molecular diagnosis, as the ultimate biomarker (Table S1).

Regarding blood and urine MD biochemical markers, no differences were observed when comparing nDNA and mtDNA patient cohorts. The most frequent cause of an impaired result was lactate and pyruvate, but they have a lack of specificity since a wide variety of genetic and environmental conditions can lead to increased lactate values [7]. GDF-15 and FGF-21 have been reported as promising biomarkers that increase diagnostic specificity, especially when muscular involvement is observed [23,25]. No differences were observed in these biomarkers when comparing clinical muscular features, but data were more predictive when considered together with histological features. Although no statistical analysis could be done due to the limited size of the series (only data from 15 patients with both FGF-21/GDF-15 analysis and muscle biopsy studies were available), a remarkable relationship between increased concentrations of these biomarkers and the presence of RRF, and blue/COX-fibers indicating muscle affectation was observed.

From a clinical point of view, the percentage of mtDNA vs. nDNA mutations in our series of patients was relatively balanced, although it is well known that mtDNA defects are much more frequent in adults than nDNA ones [1,19,26]. This finding is probably due to a biased selection in our patient cohort, as over the past few decades it has become easier to identify mutations in mtDNA than in nDNA. Muscular involvement was a prominent feature in the studied patients, which is similar to other reported series [1]. The higher frequency of muscular signs in the mtDNA group may be explained by the older age of this group when compared with nDNA patients, some of whom experienced disease onset during the newborn period or infancy with a fatal outcome. This fact could also explain the lack of differences in survival between these two patient groups, since some mtDNA patients were diagnosed several years ago, while most of the nDNA were recently identified, having a shorter follow-up period.

Histological features in MD patients have been extensively reported [4]. New technological developments have been recently published that are relevant to MD diagnosis, especially methods related to a sensitive quadruple immunofluorescent technique which enables the accurate quantification of key OXPHOS protein abundance and porin in individual myofibers [27]. Here, we evaluated only the classical biomarkers, since the cohort was recruited over the last 25 years and no immunofluorescence studies were available for older patients. COX/SDH histochemistry is the standard method used to assess mitochondrial respiratory chain function in muscle cryosections. The activities of the partially mtDNA-encoded complex IV (COX) and the fully nuclear-encoded complex II (SDH) [28] are seen as a mosaic reduction or loss of COX activity with preserved SDH activity (blue fibers), indicative of an underlying mtDNA-related abnormality. RRF reflects mitochondrial replication to compensate for energy failure [28]. Our mtDNA patients showed a higher percentage of alterations in RRF and blue fibers when compared with the nDNA group. Conversely, SDH staining alterations were higher in the nDNA group of patients, especially with regards to SDH-positive vessels. In any case, there was a trend of gradually increasing proportions of patients with mitochondrial histopathological features as their age increased [26,28].

Pathological features in infants were studied; however, it is difficult to demonstrate major alterations in muscle for this group, and the lack of RRF/blue fibers in children below the age of 5 years makes a challenging diagnosis [26,28]. In our infant cohort, only one patient with aTK2 mutation showed RRF/blue fibers, confirming these previous observations. The most frequent alteration found in this cohort was COX-/COX reduction and SDH-positive blood vessels. Regarding COX studies, it has been proposed that >2% COX-fibers in children may be indicative of MD [4]. In non-mitochondrial myopathies in infants, only COX reduction (but no COX-fibers) was detected in four cases. The presence of necrotic fibers (MDC1A) and nemaline rods, the latter causing a disrupted sarcomere structure, could explain these findings. SDH reduction was observed only in the MD group, suggesting that it might have a good specificity as a biomarker for MD diagnosis in infants, but no high sensitivity (only 5 MD cases out of 17 presented this pathological feature). SDH-positive vessels were frequently detected both in MD and non-MD patients (Figure 2), and it should be considered an unspecific finding, at least in infants. An interesting finding was the higher presence of increased lipids in MD infants compared to non-MD patients. Although this feature cannot be considered specific of MD (other lipid storage myopathies can present it in greater amount than MD), it could be an additional biomarker for MD pathological diagnosis in infants [4,29].

Despite the increasing use of NGS and other omics approaches for unraveling novel mitochondrial genes, current clinical practice for investigating MD essentially involves a multipronged approach that includes clinical assessment, metabolic screening, imaging, and pathological, biochemical and functional testing to guide molecular genetic analysis [4]; the latter should be considered as the unique real biomarker for diagnosis of MD. Recently, Morava criteria were revisited, and it was demonstrated to be a useful tool for facilitating the diagnostic workflow for MD [1]. In fact, there is still a notable percentage of patients whose diagnosis is missing. Our results support, as in other reports, that NGS techniques have substantially improved the diagnostic yield, especially for nDNA mutations, but thorough clinical/phenotypical investigation is still required.

Present and future directions: The application of NGS techniques in recent years has remarkably improved the diagnostic yield in MD. In this work, we report on our retrospective experience in the diagnosis of MD pediatric patients by applying classical diagnostic approaches. However, since NGS techniques are changing the diagnostic paradigm in genetic diseases in general, revisiting the diagnostic protocols seems necessary. At present, non-invasive approaches are mandatory in the first-line diagnosis of MD. A deep clinical investigation together with imaging and other complementary test support, metabolic testing (in blood and urine) and DNA collection for NGS analysis is advisable, avoiding invasive procedures. Depending on the results after this first line diagnosis, invasive approaches may be considered as a second-step, such as investigations in muscle/skin biopsies to interrogate mitochondrial function. This should be recommended only in cases with uncertain clinical and molecular associations (new phenotypes, new mutations of uncertain significance, and especially when new genes are candidates as a cause of MD). These kinds of approaches are supported by reputed authors in the field [1], but in all likelihood in near-future, the NGS molecular diagnosis will be so effective that it will be able to replace some of the diagnostic practices that are carried out today.

## 5. Conclusions

In conclusion, we report on the disease characteristics of a large series of pediatric MD patients, where muscular involvement both at clinical and histopathological levels was prominent, especially in those harboring mutations in mtDNA genes. The fast technological changes experienced in recent years regarding NGS strongly advise to revisit the current diagnostic protocols in clinical and laboratory settings.

## Figures and Tables

**Table 1:** Clinical, biochemical, and histopathological data in the whole cohort of patients classified as having nuclear DNA (nDNA) or mitochondrial DNA (mtDNA) mutations. Results are expressed as the percentage of cases with impaired biomarkers (elevated concentration above normal cut-off value), percentage of patients presenting with myopathic features, and percentage of muscle biopsies with positive features. Chi-square was calculated to look for associations between the different variables listed in the left column (categorized as presence or absence) and whether the mutation was in the nDNA or mtDNA. nDNA patients showed a higher frequency of succinate dehydrogenase (SDH) reduction and SDH-positive vessels, while mtDNA patients showed a higher frequency of ragged red (RRF) and blue fibers.

Percentage (%)	Total Group (*n* = 103)	nDNA Patients (*n* = 59)	mtDNA Patients (*n* = 44)	Chi-Square
Sex (M/F)	58.3/41.7	57.6/42.4	59.1/40.9	n.s.
Exitus	41.7	40.6	44.4	n.s.
Survival (years)		6.7	11.9	n.s.
**Biomarkers**				
Lactate	79.8	81.5	77.5	n.s
Pyruvate	69.1	76.1	60.0	n.s.
Alanine	64.9	61.1	70.0	n.s
Organic acids	35.3	34.0	37.5	n.s
GDF-15/FGF-21	51.4	48.0	60.0	n.s
**Myopathy**				
CPK	28.3	37.1	16.0	n.s.
Ophthalmoplegia	23.4	15.9	33.3	n.s.
Myopathic facies	20.8	16.3	26.5	n.s.
Exercise intolerance	25.3	16.7	36.4	n.s.
Weakness	26.0	22.7	30.3	n.s.
Rhabdomyolysis	3.9	4.7	2.9	n.s.
EMG (myopathic)	19.9	18.5	23.5	n.s. *
**Histopathology**				
RRF	32.8	18.2	50.0	6.959 (*p* = 0.008)
Blue fibers	36.0	20.0	60.0	8.333 (*p* = 0.004)
COX negative	39.6	41.4	36.8	n.s.
COX reduction	50.0	51.7	47.4	n.s.
SDH reduction	22.4	34.5	5.0	5.910 (*p* = 0.015)
Positive SDH (Vessels)	65.8	85.7	41.2	8.828 (*p* = 0.004)

n.s. = non-significative; * 33.3% of nDNA and 29.4% of mtDNA patients presented a normal Electromyography (EMG) while 48.2% and 47.1% showed neuropathic features; M, male; F, female; CPK, creatine phosphate kinase; RRF, ragged red; SDH, succinate dehydrogenase; COX, cytochrome c oxidase.

**Table 2:** Patients were classified as having or not having positive histopathological features (RRF, blue or cytochrome c oxidase negative (COX-) fibers; positive/negative) and muscular involvement (ophthalmoplegia, myopathic facies, exercise intolerance, weakness, high creatine kinase (CPK) values or myopathic electromyography (EMG); yes/no). Significantly positive associations among the different variables in these groups of patients are stated. Results are expressed as % of patients with the four myopathic and the three histopathological features listed in the left column.

	**Histopathology**		**Chi-Square**
	**Positive**	**Negative**	
**Myopathy**			
Ophthalmoplegia	44.0	8.3	7.991 (*p* = 0.005)
Myopathic facies	33.3	8.3	4.547 (*p* = 0.033)
Exercise intolerance	45.8	4.5	10.148 (*p* = 0.001)
Weakness	48.0	0	14.720 (*p* < 0.0001)
	**Muscular Involvement**		**Chi-Square**
	**Yes**	**No**	
**Histopathology**			
RRF	63.6	13.8	13.609 (*p* < 0.0001)
Blue fibers	65.0	18.5	10.505 (*p* = 0.001)
COX negative	63.2	16.0	10.375 (*p* = 0.001)

## Supplementary Materials

The following are available online at http://www.mdpi.com/2077-0383/8/1/68/s1, Table S1. Main clinical, biochemical, histopathological and molecular data in the entire cohort of patients. Positive histopathology was considered when patients presented with COX negative, RRF or blue fibers. Myopathy was present when one of the following signs were present: ophthalmoplegia, myopathic facies, weakness, exercise intolerance, rhabdomyolysis or myopathic EMG. mtDNA depletions were detected in tissues, mainly in muscle. In brackets, we quoted the references where some of our patients were previously published [30,31,32,33,34,35,36,37,38,39].
