# Supplementary material for: Muscle Involvement in a Large Cohort of Pediatric Patients with Genetic Diagnosis of Mitochondrial Disease
Source: J Clin Med. 2019 Jan 10;8(1):68. doi: 10.3390/jcm8010068 (PMC6352184; doi:10.3390/jcm8010068)
Supplement: Supplementary file 1 [file jcm-08-00068-s001.pdf]

Table S1. Main clinical, biochemical, histopathological and molecular data in the entire cohort of patients. Positive histopathology was considered when patients presented with COX negative, RRF or blue fibers. Myopathy was present when one of the following signs were present: ophthalmoplegia, myopathic facies, weakness, exercise intolerance, rhabdomyolysis or myopathic EMG. mtDNA depletions were detected in tissues, mainly in muscle. In brackets, we quoted the references where some of our patients were previously published [30-39]. In the column "Role in OXPHOS", (1) correspond to genes with a primary role specific to OXPHOS biogenesis and (2) to genes with a secondary impact on OXPHOS biogenesis as well as other cellular functions, as reported by Frazier et al [19].

Case	Age (onset)	Sex	Miopathy	Histopathology	Genetic diagnosis	Genetic Group	Clinical phenotype	Role in OXPHOS [19]
1	0.01	M		Positive	AGK:c.[IVS8+1G>A];[IVS8+1G>A]	nDNA	SENGERS syndrome	Protein import/processing (2)
2	0.5	F	No	Negative	arr[hg19] 5q14.3(85425299–91114671)x1 (COX7 gene deletion)	nDNA	Encephalopathy	OXPHOS subunit (1)
3	11	M	No	Negative	BCS1L:c.[166C>T];[205C>T]	nDNA	Leigh syndrome	OXPHOS Assembly factors (1)
4	3	F	Yes	Positive	Common mtDNA deletion	mtDNA	CPEO	OXPHOS subunit (1)
5	15	M	No	Positive	Common mtDNA deletion (62%) [30]	mtDNA	Kearns-Sayre	OXPHOS subunit (1)
6	0.01	F	No	Negative	DNM1L:c.[1337G>T];[1337=] [30]	nDNA	Encephalopathy	OXPHOS subunit (1)
7	0.08	M	No	Negative	ECHS1:c.[740C>T];[146T>C]	nDNA	Leigh syndrome	OXPHOS subunit (1)
8	7	F	No	Negative	FARS2:c.[1256G>A];[(672+1_673-1)_(904+1_905-1)del]	nDNA	Encephalopathy/Spastic paraparesis	Mitochondrial morphology (2)
9	0.01	M	No		FBXL4:c.[851delC];[851delC]	nDNA	Mitochondrial DNA depletion syndrome	Metabolism of toxic compounds (2)
10	0.16	F	No	Positive	GFM1:c.[1401delA];[2011C>T] [32]	nDNA	Encephalopathy	Mitochondrial tRNA biogenesis (1)

11	0.18	M	No		HIBCH:c.[517+1G>A];[353T>C]	nDNA	Encephalopathy	Unclear function
12	0.16	M	No		NDUFAF2:c.[(?_-127)_(217+1_218-1)del)];[(?_-127)_(217+1_218-1)del)]	nDNA	Encephalopathy	Translation (1)
13	0.16	F	No	Positive	KARS:c.[650G>A];[1709C>G]	nDNA	Encephalopathy	Metabolism of toxic compounds (2)
14	6	M	No	Positive	MT-ATP6	mtDNA	NARP	OXPHOS Assembly factors (1)
15	2	F	Yes	Positive	MT-ATP6:m.8573G>A	mtDNA	Leigh/NARP	Mitochondrial tRNA biogenesis (1)
16	0.01	F	No	Negative	MT-ATP6:m.8993T>G	mtDNA	NARP	OXPHOS subunit (1)
17	0.2	M	No	Negative	MT-ATP6:m.8993T>G	mtDNA	NARP	OXPHOS subunit (1)
18	0.3	M	No	Negative	MT-ATP6:m.8993T>G	mtDNA	Encephalopathy	OXPHOS subunit (1)
19	0.3	F	No	Negative	MT-ATP6:m.8993T>G	mtDNA	NARP	OXPHOS subunit (1)
20	1	F	No	Negative	MT-ATP6:m.8993T>G	mtDNA	Leigh syndrome	OXPHOS subunit (1)
21	1	F	Yes	Negative	MT-ATP6:m.8993T>G	mtDNA	Leigh syndrome	OXPHOS subunit (1)
22	2	M	No	Negative	MT-ATP6:m.8993T>G	mtDNA	Leigh syndrome	OXPHOS subunit (1)
23	2	F		Negative	MT-ATP6:m.8993T>G	mtDNA	NARP	OXPHOS subunit (1)
24	1	M	Yes		MT-ATP6:m.9176T>C	mtDNA	Leigh/NARP	OXPHOS subunit (1)
25	0.01	F			MT-CO1:m.7258T>C	mtDNA	LEBER OPTIC ATROPHY	OXPHOS subunit (1)
26	3	F	Yes	Positive	mtDNA deletion (11041 bp)	mtDNA	Kearns-Sayre	OXPHOS subunit (1)
27	3	M	Yes	Positive	mtDNA deletion (4120 bp) [30]	mtDNA	Kearns-Sayre	OXPHOS subunit (1)
28	9	F	Yes	Positive	mtDNA deletion (60%)	mtDNA	Kearns-Sayre	OXPHOS subunit (1)
29	4	M	Yes	Positive	mtDNA deletion (600 bp)	mtDNA	Kearns-Sayre	OXPHOS subunit (1)
30	8	M		Positive	mtDNA deletion (6700 bp) [30]	mtDNA	Kearns-Sayre	OXPHOS subunit (1)
31	9	M		Positive	mtDNA deletion (7663 bp)	mtDNA	Kearns-Sayre	OXPHOS subunit (1)
32	9	F	Yes	Positive	mtDNA deletion (77%; 2434 bp) [30]	mtDNA	Kearns-Sayre	OXPHOS subunit (1)
33	16	M	Yes		mtDNA deletion (80%)	mtDNA	Kearns-Sayre	OXPHOS subunit (1)
34	1	M	No		mtDNA deletion (90%; 3600 bp)	mtDNA	Pearson	OXPHOS subunit (1)

35	0.4	M			mtDNA depletion (70%)	nDNA	Myopathy	Not solved
36	0.5	F	No	Negative	mtDNA depletion (70%)	nDNA	Mitochondrial DNA depletion syndrome	Not solved
37	1	F		Negative	mtDNA depletion (70%)	nDNA	Encephalopathy	Not solved
38	1	F	Yes	Positive	mtDNA depletion (70%)	nDNA	Encephalopathy	Not solved
39	0.01	M			mtDNA depletion (72%)	nDNA	Encephalopathy	Not solved
40	0.02	F	No		mtDNA depletion (75%)	nDNA	Mitochondrial DNA depletion syndrome	Not solved
41	2	M	No		mtDNA depletion (80%)	nDNA	Mitochondrial DNA depletion syndrome	Not solved
42	0.1	M		Negative	mtDNA depletion (84%)	nDNA	Myopathy	Not solved
43	2	M		Positive	mtDNA depletion (90%)	nDNA	Mitochondrial DNA depletion syndrome	Not solved
44	11	M			MT-ND1:m.3460G>A	mtDNA	LEBER OPTIC ATROPHY	OXPHOS subunit (1)
45	14	M	No		MT-ND1:m.3460G>A	mtDNA	LEBER OPTIC ATROPHY	OXPHOS subunit (1)
46	4	M		Negative	MT-ND1:m.3547A>G; MT-ND5:m.11177C>T	mtDNA	NARP	OXPHOS subunit (1)
47	5	M			MT-ND1:m.4216T>C	mtDNA	Optic atrophy syndrome	OXPHOS subunit (1)
48	7	M	Yes		MT-ND5:m.13513G>A	mtDNA	Leigh/NARP	OXPHOS subunit (1)
49	14	F	No	Positive	MT-ND5:m.13513G>A	mtDNA	MELAS	OXPHOS subunit (1)
50	11	M	No		MT-ND6:m.14495A>G	mtDNA	LEBER OPTIC ATROPHY	OXPHOS subunit (1)
51	13	F	Yes	Positive	MT-TA:m.5624T>C	mtDNA	Myopathy	Mitochondrial tRNA biogenesis (1)
52	8	M	No		MT-TK:m.8344A>G (90%)	mtDNA	MERRF	Mitochondrial tRNA biogenesis (1)
53	5	F		Negative	MT-TK:m.8363G>A [22]	mtDNA	MERRF	Mitochondrial tRNA biogenesis (1)
54	2	M	Yes	Positive	MT-TL1:m.3243A>G	mtDNA	MELAS	Mitochondrial tRNA biogenesis (1)

55	2	F	Yes		MT-TL1:m.3243A>G	mtDNA	MELAS	Mitochondrial tRNA biogenesis (1)
56	4	M	Yes	Positive	MT-TL1:m.3243A>G	mtDNA	MELAS	Mitochondrial tRNA biogenesis (1)
57	5	M	Yes		MT-TL1:m.3243A>G	mtDNA	MELAS	Mitochondrial tRNA biogenesis (1)
58	6	F	No		MT-TL1:m.3243A>G	mtDNA	MELAS	Mitochondrial tRNA biogenesis (1)
59	6	M	Yes		MT-TL1:m.3243A>G	mtDNA	MELAS	Mitochondrial tRNA biogenesis (1)
60	10	M	No	Positive	MT-TL1:m.3243A>G	mtDNA	MELAS	Mitochondrial tRNA biogenesis (1)
61	13	M	No	Positive	MT-TL1:m.3243A>G [22]	mtDNA	MELAS	Mitochondrial tRNA biogenesis (1)
62	10	F	Yes		MT-TL1:m.3252A>G	mtDNA	MELAS	Mitochondrial tRNA biogenesis (1)
63	15	F			MT-TS2:m.12237delC	mtDNA	MERRF/MELAS OVERLAP SYNDROME	Mitochondrial tRNA biogenesis (1)
64	2	M	No	Positive	MT-TV:m.1643A>G	mtDNA	Encephalopathy	Mitochondrial tRNA biogenesis (1)
65	3	F		Negative	Multiple mtDNA deletions	nDNA	Encephalopthy	OXPHOS subunit (1)
66	5	M	Yes	Negative	Multiple mtDNA deletions	nDNA	CPEO	OXPHOS subunit (1)
67	2	M	Yes		NDUFS2:c.[422A>G];[?]	nDNA	Leigh syndrome	OXPHOS subunit (1)
68	6	F	No		NDUFS2:c.[422A>G];[?]	nDNA	Leigh syndrome	OXPHOS subunit (1)
69	0.5	F	No	Negative	NDUFS4:c.[291delG];[291delG] [33]	nDNA	Leigh syndrome	OXPHOS subunit (1)
70	1	F	No		NFU1:c.[545+5G>A];[622G>T] [34]	nDNA	Encephalopathy	Fe-S cluster biogenesis (2)
71	0.58	F	Yes	Positive	OPA1:c.[1710T>G];[1710=]	nDNA	Optic atrophy plus syndrome	Mitochondrial morphology (2)
72	0.13	F	Yes	Positive	OXA1L:c.[500_507dup];[620G>T] [35]	nDNA	Myopathy	OXPHOS Assembly factors (1)

73	2	M	No	Negative	PDHA1:c.[1143_1144ins24]	nDNA	Leigh syndrome	Krebs cycle and metabolism (2)
74	5	F	No		PDHA1:c.[409G>C];[409=]	nDNA	Encephalopathy	Krebs cycle and metabolism (2)
75	0.33	F	No	Negative	PDHA1:c.[498C>T];[498=]	nDNA	Leigh syndrome	Krebs cycle and metabolism (2)
76	1	M	Yes		PDHA1:c.[787C>G]	nDNA	Leigh syndrome	Krebs cycle and metabolism (2)
77	1	M	Yes	Negative	PDHA1:c.[832G>A] [34]	nDNA	Encephalopathy	Krebs cycle and metabolism (2)
78	1	M	Yes	Positive	PDHB:c.[301A>G];[42+1G>A] [36]	nDNA	Leigh syndrome	Krebs cycle and metabolism (2)
79	1	F	Yes	Negative	POLG:c.[2591A>G];[3649G>C] [8]	nDNA	Encephalopathy	Mitochondrial DNA homeostasis (1)
80	1	M	No	Negative	POLG:c.[911T>G];[2663G>A]	nDNA	Encephalopathy	Mitochondrial DNA homeostasis (1)
81	11	M	Yes	Positive	POLG:c.[911T>G];[911T>G]	nDNA	Myopathy	Mitochondrial DNA homeostasis (1)
82	0.02	M	No	Negative	PUS1:c.[1236+7G>A];[1236+7G>A]	nDNA	Leigh syndrome	Mitochondrial tRNA biogenesis (1)
83	2	F	No		RARS2:c.[442A>G];[34C>T]	nDNA	Ataxia	Mitochondrial tRNA biogenesis (1)
84	1	M	No	Negative	SLC19A3:c.[1079dupT];[980-14A>G] [37]	nDNA	Leigh syndrome - Biotin responsive basal ganglia disease	Metabolite transport (2)
85	4	M	No		SLC19A3:c.[68G>T];[68G>T] [37]	nDNA	Leigh syndrome - Biotin responsive basal ganglia disease	Metabolite transport (2)
86	4	F	No		SLC19A3:c.[74dupT];[980-14A>G] [37]	nDNA	Leigh syndrome - Biotin responsive basal ganglia disease	Metabolite transport (2)

87	13	M	Yes		SLC19A3:c.[74dupT];[980-14A>G] [37]	nDNA	Leigh syndrome - Biotin responsive basal ganglia disease	Metabolite transport (2)
88	2	F	No	Negative	SUCLA2:c.[1048G>A];[1049G>T] [38]	nDNA	Leigh syndrome	Nucleotide pools (1)
89	5	M	Yes		TAZ:c.[94A>C]	nDNA	Barth syndrome	Lipid modification (2)
90	2	M	Yes	Positive	TK2: c.[276dupA];[730_732delAAG]	nDNA	Myopathy	Nucleotide pools (1)
91	1	M	Yes		TK2:c.[360_361delGCinsAA];[575G>A] [39]	nDNA	Myopathy	Nucleotide pools (1)
92	2	M	Yes	Positive	TK2:c.[360_361delinsAA];[360_361delinsAA]	nDNA	Miopathy	Nucleotide pools (1)
93	1	M		Positive	TK2:c.[388C>T];[623A>G]	nDNA	Miopathy	Nucleotide pools (1)
94	3	M	Yes	Positive	TK2:c.[388C>T];[633A>G]	nDNA	Myopathy	Nucleotide pools (1)
95	1	F	Yes		TK2:c.[602-6del];[529G>T]	nDNA	Miopathy	Nucleotide pools (1)
